# Bacteriological Contamination of Drinking Water Supply from Protected Water Sources to Point of Use and Water Handling Practices among Beneficiary Households of Boloso Sore Woreda, Wolaita Zone, Ethiopia

**DOI:** 10.1155/2020/5340202

**Published:** 2020-04-12

**Authors:** Matusala Gizachew, Amha Admasie, Chala Wegi, Etagegnehu Assefa

**Affiliations:** ^1^Plan International Ethiopia, Boloso Sore Woreda Project Area, Wolaita Zone, Addis Ababa, Ethiopia; ^2^Bahir Dar University, School of Public Health, Bahir Dar, Ethiopia; ^3^Wolaita Sodo University, School of Public Health, Wolaita Sodo, Ethiopia; ^4^Wolaita Sodo University, Department of Chemistry, Wolaita Sodo, Ethiopia

## Abstract

The quality of drinking water is a powerful environmental determinant of health. Water becomes contaminated with faecal material due to inadequate protection of the source, unhygienic practices of the community at the source, and poor household handling practices. The objective of this study was to assess the level of bacteriological contamination of drinking water supply from protected water sources to point of use and water handling practices among beneficiary households of Boloso Sore woreda, Wolaita zone, Ethiopia. A cross-sectional survey and bacteriological analysis of water were conducted in January 2019. The study included 545 households for water handling practices, and 75 samples from stored water from households and eighteen water sources were included for faecal coliform test. Data were analyzed using SPSS v21.0. Descriptive and logistic regression statistical models were used. Sixty percent of shallow wells, 60% of protected hand-dug wells, and 25% of protected on-spot springs were found positive for faecal coliform. In general, 44% of water source samples and 91% of household water samples were positive for faecal coliform. In general, 38% of households were practicing unsafe water handling practices. High school and above level of education (AOR = 3.37, 95% CI: 1.03, 11.57), getting higher monthly income (AOR = 2.37, 95%CI: 1.96, 5.85), households with small family size (AOR = 1.81, 95% CI: 1.15, 2.83), frequency of water collection twice a day (AOR = 2.88, 95% CI:1.56, 5.33), and presence of water payments (AOR = 0.42, 95% CI: 0.24, 0.72) were significantly associated with water handling practice. Unsafe water handling was a common practice in the study area, and water sources and household water storage were not free of faecal coliform, indicating noncompliance with the World Health Organization water quality guideline. Hence, capacity building is mandatory for the protection and management of water sources and safe water handling practices in the household and community.

## 1. Introduction

Safe drinking water is one of the basic necessities for human beings. However, billions of people in the world do not have access to safe drinking water, appropriate sanitation, and hygiene in developing countries [1]. The quality of drinking water is a powerful environmental determinant of health and continues to be the foundation for the prevention and control of waterborne diseases.

There are several variants of the faecal-oral pathways of water-borne disease transmission. These include contamination of drinking water catchments (e.g., by pathogens of faecal origin, human or animal faeces), water within the distribution system, or stored household water as a result of unhygienic handling [2, 3]. Contamination can occur as the water is taken out of the storage container. Hands and utensils may come into contact with the water [2].

Current World Health Organization (WHO) guidelines for drinking water quality support efforts to ensure safe collection, treatment, and storage of drinking water. The absence of indicator organisms in drinking water indicates its bacteriological quality and does not pose health risk if consumed [4]. Traditionally, total coliform bacteria have been used to indicate the presence of faecal contamination. An exception is *Escherichia coli* (*E. coli*), a thermotolerant coliform, and the most numerous of the total coliform group found in animal or human feces, rarely grows in the environment and is considered the most specific indicator of faecal contamination in drinking water [2]. The presence of *E. coli* provides strong evidence of recent faecal contamination and is used to estimate disease risk [2]. *E. coli* bacteria as a microbial water quality indicator should be zero per 100 ml water for drinking purpose [4]. The majority of the drinking water sources are either of unacceptable quality or grossly polluted water [5]. Ensuring the safety of drinking water is a continuous process which requires the monitoring of multiple microbiological and chemical parameters [6, 7].

In Dire Dawa, Ethiopia, water handling practice in the Adada village shows that the most commonly preferred type of water collection container was jerry can which accounted for 59.37% followed by clay pots 40.63%. In relation to the way that the respondents withdrew water from containers, 8 (6.25%) preferred pouring and the remaining 93.75% by dipping [8].

In Tehuledere woreda, Amhara region, Ethiopia, water handling practice shows that 54.7% of the households were found to collect water in clay pots and 44.7% in jerry can. The majority (92.7%) do have cover for their storage containers. Drawing of water from storage containers was carried out by dipping in 72.0% and pouring in 28.0% of cases [9].

Water quality assessments based on water source typology indicated that the quality of drinking water is highly influenced by water source types [10]. In Tamale, Ghana, research shows that out of 40 water samples examined from storage vessels, 55.0% were faecally contaminated. Furthermore, water stored in jerry cans was found to have significantly better bacteriological quality than water stored in clay pots (*P* < 0.05). The concentration of faecal coliform significantly reduced in those households using pouring than dipping (*P* < 0.05) [11].

A study in rural areas of Ethiopia found that about 74% and 58% of the water samples from water sources and household storage were positive for *E. coli* [6]. In Jimma zone, Oromia region, Ethiopia, among the 15 protected well water samples analyzed, 40% had bacterial count below 10 CFU/100 ml and 26.67% were free of faecal coliforms. Sixty percent of protected springs were free from faecal coliforms and 46.67% of these samples had thermotolerant coliforms (TTC) count less than 10 CFU/100 ml [12].

In Farta woreda, Amhara region, Ethiopia, of the total 30 water samples from protected water sources, 90% were above the standard limit of the WHO. Of the total 24 water samples collected from protected wells, 12.5%, 16.7%, 41.7%, and 20.85% had *E. coli* concentrations ranging from ≥1000, 100–1000, 10–100, and 1–10, respectively. Similarly, from the total 6 water samples obtained from protected spring, 83.3% had *E. coli* ranging from 10 to 100/100 CFU/100 ml of water [13].

In the same woreda with a different study, all sampled (a total of 90 households) water containers had *E. coli*. In case of risk classification, 33.3% and 8.3% of protected well water samples had very high and low sanitary risk scores for *E. coli*, respectively [14].

In Wondo Genet woreda, southern Ethiopia, a study found that among 28 randomly selected water sources (14 on-spot springs and 14 dug wells fitted with hand pump), 25% of water sources were contaminated with *E. coli* while more than 85% the samples were contaminated with total coliforms [10]. A study conducted in Bahir Dar city, Amhara region, Ethiopia, showed, in the case of TTC of the household, 16 (45.7%) and 14 (40%) had counts ranging from 10 to 100 and 1.01 to 9.99 CFU/100 ml, respectively [15]. Narrow-mouthed storage containers are the safest method of water storage, but it may be often difficult to properly clean them after emptying [10].

The national and the regional information on the water quality status and household water handling practices are not well studied, and there is also a limited microbiological quality of water in rural areas of Ethiopia, especially in the study area, Boloso Sore woreda. Hence, the present study aimed at assessing the level of bacteriological contamination of drinking water supply from protected water sources to point of use and water handling practices among beneficiary households of Boloso Sore woreda, Wolaita zone, Ethiopia.

## 2. Materials and Methods

Boloso Sore woreda is situated in Wolaita zone of Ethiopia with a population of 219,649. The woreda consists of 5 urban kebeles, 27 rural kebeles, 8 health centers, and 28 rural health posts. Woreda has coverage of health service (92%), improved latrine (32%), and improved functional water supply (49.6%). There are 56 protected springs, 63 shallow wells, 57 protected hand-dug wells, 7 boreholes, and unprotected hand-dug well for all domestic water uses in the woreda (Boloso Sore woreda 2018/19 report). The study was conducted in Ethiopian dry season of January 2019. During this dry season, farmers are completing their agricultural activities; the community uses water intensively for domestic purpose, and the water volume gets declined. There is no irrigation practice in the community; however, cattle herds are common in the study area, and cattle use these water sources for drinking, which can be a risk of water source contamination. Most water sources have fences, but not strong to protect the entrance of cattle and children.

A cross-sectional study was conducted in randomly selected rural households that benefited from functional protected water sources of Boloso Sore woreda. Mother/adult persons were respondents. Households that benefit from functional protected water sources that are functional in the time of the study were included, while households that benefited from other sources were excluded.

Sample size was calculated by using a single population proportion formula with 95% confidence interval, 5% margin of error. Since there were no previous related studies conducted in the study area, the assumption of 50% population proportion was taken for unsafe water handling practice with a design effect of 1.5 and 10% of nonresponse rate. Therefore, the sample size was 578.

Seven kebeles were selected randomly (Tokisa Godo, Dolla, Bassa Gofara, Afama Bancha, Chama Hembecho, Sore Homba, and Achura). Water quality test was conducted from 18 water sources and 75 household storage materials. These 75 households were a part of assessment of household water handling practices. For better analysis and comparison of the level of contamination from source to point of use, the same households are applied for the assessment of water handling practice. In general, sample sizes for water handling practice were 545 HHs, for bacteriological quality tests were 75 HHs, and 18 water sources.

From the total 27 rural kebeles found in the woreda, 7 kebeles (25%) were selected randomly. Since the study was conducted on protected water source beneficiary households, 18 protected water sources were purposively selected based on the scheme type shallow well (SW), hand-dug well (HDW), and spring (SP) among 65 protected water sources in selected seven kebeles. Then, 578 households were proportionally distributed to each selected scheme. A list of beneficiary households was found in the registration book of each scheme.

Purposively, 18 protected water sources (5 hand-dug wells, 8 spring, and 5 shallow wells) were included from the total of 65 functional protected water sources found in 7 selected kebeles of the woreda. Under each selected water source, five households were randomly selected for bacteriological water analysis to observe contamination variation from household to household and source to households. The sample from the source was taken correspondingly with the household water sample after asking the inhabitants where they fetch water during the time of household water sample collection.

**Figure 1:**
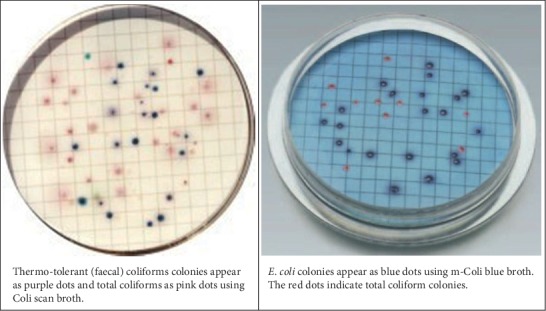
Different indicator bacteria colonies. (a) Thermotolerant (faecal) coliform colonies appear as purple dots and total coliforms as pink dots using Coliscan broth. (b) *E. coli* colonies appear as blue dots using m-Coli blue broth. The red dots indicate total coliform colonies.

**Figure 2:**
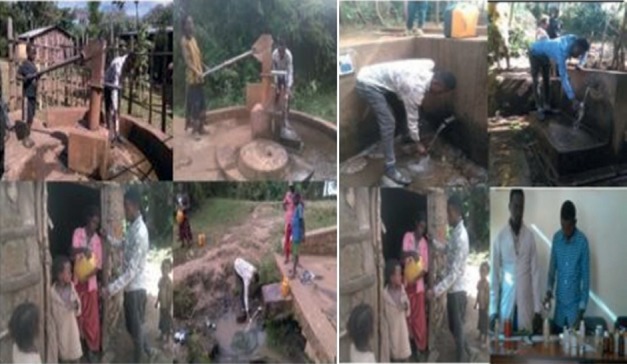
Water sampling and quality testing at a water quality laboratory.

For the analytical study, water samples were collected from the source spring boxes, from the distribution points (faucets, bono in Amharic language) in selected clusters, and from the households by applying systematic random sampling technique. For bacteriological analysis, samples were collected in sterilized plastic bottles (100 ml). Water samples were collected after sterilizing the taps with ignited cotton wool soaked in alcohol aseptically. The water samples were transported to the laboratory by maintaining the cold chain system. All samples were analyzed for faecal coliform count within 4 hours of sample collection. Faecal coliform (FC) enumeration was carried out using membrane filtration techniques in which 100 ml of water sample was filtered through the membrane filter (Millipore 45 *μ*m). Membrane lauryl sulfate medium that was dispensed onto the absorbent pad was used for bacterial growth medium. Then, the membrane filter through which the water samples were filtered was placed on the membrane lauryl sulfate medium in aluminum Petri dish and incubated at 44 ± 0.5°C for 12–16 hours. All the analysis was carried out by using the Oxfam deluge test kit (Robens Centre for Public and Environmental Health, University of Surrey Guildford, United Kingdom) (see Figures 1 and 2).

Data collection method was done by using questionnaire, observation with checklist, and taking water samples. The questionnaires were originally developed in English and then translated to the local language (Wolaitigna). The questionnaire was pretested in 29 households of Yukara kebele to check the consistency. The water samples from the source and household storage were collected by trained laboratory technicians.

### 2.1. Safe Water Handling Practice Criteria

It was measured using 14 safe handling practice criteria: washing hands before for collecting water, washing and rinsing practice of container before collection, covering for water collection container, type of water storage container, method of drawing water, putting drinking cups in safe place, separate drinking cups, household water treatment practice, container washing materials, type of water collection container, duration of washing storage container, cover for storage, how long you stored water, and cleanness of water storage (adopted from different literature studies).

### 2.2. Good Water Handling Practice

Good water handling practices are practices that fullfil above the average value (>8) of the safe water handling practice criteria.

Training was given for data collectors and supervisors for two days on the procedures, techniques, and ways of data collection. Data collectors conducted pretests. All water samples were collected by trained laboratory technicians in strict supervision and procedure. All sampling bottles were appropriately labeled, and the samples were collected using standardized drinking water sampling techniques. The collected water samples kept in icebox during transportation put at 4 degree Celsius before analysis in the laboratory. Before analysis, sterilization of required laboratorial equipment and culture medium was carried out. Moreover, to ensure the validity of the analysis, blank samples were analyzed following the same procedure. Water quality analysis guideline, protocol, and quality control were used.

The coded data were entered into Epi Info version 3.5.1 and exported to SPSS version 21 software for statistical analysis. Descriptive statistics, OR with 95% CI, *P* value, and multicollinearity tests were done. Hosmer and Lemeshow goodness-of-fit test was done to assess the fitness of the model. The bivariate analysis was done for safe and unsafe water handling practices. The independent variable (water handling practice) with *P* value ≤0.25 during bivariate analysis was entered into multivariable analysis model. Statistical significance was declared at *P* value <0.05.

Ethical clearance was obtained from the Institutional Ethical Committee of Wolaita Sodo University. After thoroughly discussing the ultimate purpose and method of the study, a written consent was obtained from all respondents. The privacy and confidentiality were maintained during the interview. Participation in the study was based on willingness and the participants had the full right not to participate.

## 3. Results and Discussion

### 3.1. Results

This study was conducted in 545 households with a response rate of 94.3%. Among the respondents, 461 (84.6%) of them were females, 781 (93.6%) of them were married, and 345 (63.3) of them no formal education. The mean ages of the respondents were 33 (SD ± 1.285) years and most (177, 32.5%) of the respondents were in the range of >35. The mean family size of the respondent households was 5.8 with SD ± 1.624. Four hundred sixty-one (84.6%) respondents were housewives. Ninety-three percent of the household's average monthly income was less than 500.00 Ethiopian Birr (ETB) (Table 1).

Households were using different water sources; 163 (29.9%), 123 (22.6%), and 259 (47.5%) of them were benefited from shallow well, protected hand-dug well, and protected spring, respectively. The time required to fetch water was calculated; 447 (82%) fetched water in the distance of <30 minutes. The most commonly preferred type of water collection container was jerry can (540, 99.1%). Only 48 (37.5%) of the respondents cleaned their containers before collection and 462 (84.8%) covered the collection container during transportation (Table 2).

Among the study inhabitants using separate containers to store water, 471 (86.4%) households preferred jerry can and 498 (91.4%) of them washed storage containers every time before filling. Households used different methods for withdrawing water from containers, and 493 (90.5%) of the respondents preferred pouring. Among those respondents, 373 (68.4%) used separate cups for drinking purpose. In regard to the placement of drinking utensils, 289 (53%) put on the table, 114 (20.9%) hung on the wall, and 142 (26.1%) placed on the floor, respectively. The currently employed rinsing materials used by the collectors were water, soap or detergent, plant leaves, and ashes/other materials like sand in 25.5%, 21.8%, 35.6%, and 17.1%, respectively (Table 3).

Among 18 water sources examined during data collection, 10 (56%) of the water sources had <1 CFU/100 ml and the rest (8, 44%) of the sources had above 1 CFU/100 ml. The sources of household water in study areas were mainly shallow well, protected hand-dug well, and protected springs. Regarding the quality of water, 60% of shallow well water, 60% of hand-dug well, and 25% protected spring were positive for *E. coli* (Table 4).

Of the total 5 water samples collected from protected hand-dug wells, 2 (40%), 2 (40%), and 1 (20%) had *E. coli* concentrations ranging from <1, 1–10, and 11–50, respectively. Similarly, from the total 8 water samples obtained from protected springs, 6 (75%) had *E. coli* concentration <1, 1 (12.5%) had 1–10, and the rest had 11–50, *E. coli*/100 ml of the water sample. Water samples were taken from 75 households in their water storage containers. Hence, of the 75 water samples examined from collection vessels, 68 (91%) were faecal contaminated. Among the faecal contaminated households, 6 households, 21 households, and 38 households had *E. coli* concentration ranging from >100, 11–50, and 1–10, respectively (Table 5).

### 3.2. Factors Associated with Water Handling Practice

On the factors that affect household's water handling practices, the bivariate logistic analysis was conducted to identify the statistically significant relation between household water handling practices and behavioral factors. Among variables run for binary logistic regression, only education level of respondents, monthly income, family size, water collection per day, presence of payment for water, type of cleansing material for water containers, and prior knowledge of water treatment were significantly associated with water handling practice during a multivariate logistic regression at *P* value <0.05.

High school and above level of education were 2.37 times more likely to practice safe water handling practice compared to illiterates (AOR = 3.37; 95% CI: 1.03, 11.57). Getting higher monthly income was 2.37 times more likely to practice safe water handling (AOR = 2.37; 95% CI: 1.96, 5.85). Households with small family size were 1.81 times more likely to practice safe water handling (AOR = 1.81; 95% CI: 1.15, 2.83). Frequency of water collection twice a day was 2.88 times more likely to practice safe water handling than collecting three times a day (AOR = 2.88; 95% CI:1.56, 5.33). Presence of prior knowledge of water treatment practice was 2.40 times more likely to practice safe water handling (AOR = 2.40; 95% CI: 1.52, 3.79). Presence of water payments hinders to practice safe water handling (AOR = 0.42; 95% CI: 0.24, 0.72) (Table 6).

### 3.3. Discussion

Among the observed 545 households, 71.2% practiced safe water handling practices at their home. This finding is agreed with the same study conducted at Jigjiga, Ethiopia, which showed 91.6% of the respondents use jerry cans for water collection [16].

Washing and rinsing practice of containers before collection and cover of collection container was observed among 91.4% and 84.8% of respondents. During transport from distribution points to their respective homes, about 84.8% of the collectors covered their filled containers. The finding is consistent with the same study conducted at Sidama zone, Bona woreda, South Ethiopia; covering of the collection containers practices was found to be 74.7% [17] which is lower than the study conducted in Kola-Diba town, Gondar, Ethiopia (96%) [17] and higher than the study conducted in Dire Dawa city administration: Adada and Legebira villages (37.5%). The currently employed rinsing materials used by the collectors were water, soap or detergent, ash, plant leaves, and other materials like grasses in 25.5%, 21.8%, 11.4%, 35.6%, and 5.7% of the cases, respectively, which is lower than the study conducted at Kola-Diba town, which showed 29.1% using water, 46.1% using soap, and 0.7% using other materials [17].

Pouring through tilting the vessel or through the use of a clean, special utensil for this purpose only are the safe methods to draw water from containers for use. Water transfer by pouring shows a significant reduction in the concentrations of faecal coliform as dipping practice increased the risk of contamination by unclean cups and through hand contact [18]. The finding is almost agreed with the study conducted at Jigjiga, which showed 86.6% of the surveyed households use the pouring practice [16], and this was almost higher when compared with studies conducted in Zambia with 80% and in South Wollo with 72% of the households dipping out from the container [9]. The reason for these many differences may be due to the use of narrow-necked clay pots and jerry can, which is inconvenient for dipping in the study.

After use, drinking utensils were mostly kept on the table by 53% of the respondents while 26.1% left on the floor and 20.9% hanged it on the wall. The same study done at Jigjiga showed 62.2% of the households put on the table, 4.6% hanged on the wall, and 30.7% put on the floor [16], which is lower than the study conducted at Kolladiba town, which showed 75.5% put on the table, 9.7% put on the floor, and 4.8% hanged on the wall, respectively [17], which is higher than the same study held in Dire Dawa Adada and Legebira villages [8] and higher than the same study conducted at Tehuledere, Northeast Ethiopia, which showed only 51 (26.6%) of the households put water drawing utensils on tables and shelves while the majority (73.4%) put it on the floor, or hang it on the wall or leave it inside the container [9].

The current study indicates that protected hand-dug wells and shallow wells had significantly more *E. coli* (60% of tested samples) as compared to protected springs (25% of tested samples). The same study held in Northwest Farta woreda, Amhara region, Ethiopia, showed 83.3% of sample springs and 91.7% protected wells were positive for *E. coli* [14]. This finding is agreed with the study conducted at Fogera and Mecha woredas of North Gonder, Ethiopia, which showed 73.77% of community water source samples were contaminated with *E. coli*. Of them, 58.62 were from protected dug wells [5].

Many studies used total coliforms, faecal coliforms, or *E. coli* as faecal contamination indicator, reflecting available water testing technology in most developing countries [1] including Ethiopia. In this study, among 18 protected water sources, 10 (56%), 5 (28%), 2 (11%), and 1 (5%) were excellent, acceptable, unacceptable, and grossly polluted, respectively. The proportion of 1–10 and 11–50 CFU/100 ml water count is significantly higher for protected hand-dug well and shallow well, but it is lower for protected spring. The variation might be the protected springs are continuously openly flowed and easy to wash. This is also supported by a finding from Farta woreda [14]. The study in Serbo town, southwest Ethiopia, showed that fifty percent had faecal coliforms, of these 35.7% had *E. coli* [19]. The contamination of these water sources might be due to poor source protections. The current study shows that all the samples were collected from protected source water benefiting households and at source level 44% of source water sample and at household level 68 (91%) positive for thermotolerant coliform. The bacteriological analysis of water at household storage containers in the current study revealed that 91% of samples were contaminated with *E. coli*.

This finding is agreed with the sample from store at Farta woreda, which showed 100% household storage samples were contaminated with *E. coli* [14, 20]; a similar study conducted at Ghana Temale Metropolis found that 83% of household samples were positive for *E. coli* [11], and this finding was in compliance with the study conducted at Kolladiba town of Ethiopia, which showed that 32.5% water samples from household storage containers were found to be positive for faecal coliforms [17]. Similarly, a study conducted in Bona woreda of southern Ethiopia and Jimma zone of southwest Ethiopia showed that majority of water samples taken from household storage containers were not in compliance with the WHO guideline value of 0 CFU/100 ml [12, 21, 22]. The poor water quality observed in storage containers might be due to the poor handling practice of the inhabitants in collection and storage. The behavioral and hygienic practices of the community might also be contributing to this high load of indicator organisms.

The bacteriological quality analysis of household water samples of the study area shows that, of the total 75 households container, 91% had contaminated with *E. coli*. Among them 7 (9%), 38 (51%), 21 (28%), 3 (4%), and 6 (8%) had *E. coli* concentration range <1, 1–10, 11–50, 51–100, and >100, respectively. The *E. coli* detected in this study indicates that there might be higher human involvement in the contamination of water sources and poor sanitation of the water supply system. The household water contamination might be highly attributed to the low level of hygiene and poor water handling practices. This is supported by a finding from Bahir Dar, Ethiopia, that reported coliform contamination of household water is associated with poor water handling practice [15].

In the study area, it has been observed that faecal coliforms were more in storage containers water than that from sources, suggesting that contamination may occur either due to bacterial regrowth or during collection, transport, storage, and drawing of water [1]. This study has limitations due to collecting water samples in one time only, in which seasonal change could not be considered.

High school and above level of education were 2.37 times more likely to practice safe water handling practice compared to illiterates (AOR = 3.37; 95% CI: 1.03, 11.57). This study is in line with the study conducted by Fenet Belay Daba and Alemayehu Oljira Wolde, 2016, indicated the educational level of a house head is positively related with the per capita daily water consumption and water handling practice [23], and those whose heads had attained postprimary education (adjusted OR = 1.48; 95% CI (1.02–2.17)) [24]. This is because education level determines the ability to decide to live a better way of life; hence, households lead by high school and above level of education were practicing good water handling practice.

Getting a higher monthly income was 2.37 times more likely to practice safe water handling (AOR = 2.37; 95% CI: 1.96, 5.85). This is also consistent with the study [23] which revealed that there is a positive relationship between monthly income and water handling practice. This is the fact that household income affects the quality and access to clean water in different ways. Income and water quality have a direct relationship. Those households with better income could have better quality water sources and are able to manage its quality in their house.

Frequency of water collection twice a day was 2.88 times more likely to practice safe water handling than collecting three times a day (AOR = 2.88, 95% CI: 1.56, 5.33). This study is concurrently agreed with the study that showed that water collection container and water handling practices also affect household water quality [25]. This is because more frequently collecting water may lead to contamination of water.

Households with small family size were 1.81 times more likely to practice safe water handling (AOR = 1.81; 95% CI: 1.15, 2.83). The number of family size has an impact on the water access, quality, and handling practice in the home. Hence, smaller family size is manageable to access quality water and to practice safe water handling. Presence of prior knowledge of water treatment practice was 2.40 times more likely to practice safe water handling (AOR = 2.40; 95% CI: 1.52, 3.79). This study is in line with a study conducted Ssemugabo et al.; their respondents were asked whether they knew the dangers associated with drinking unsafe water, majority (97.2%, 384/395) of the participants said they did and (61.8%, 244/395) indicated that boiling drinking water was key to preventing diarrheal diseases [24]. It is linked to access of information on how to keep the quality of water in households and in the community. Local health extension workers provide health education and promotion activities to the community regularly on water quality.

Presence of water payments hinders to practice safe water handling (AOR = 0.42; 95% CI: 0.24, 0.72). It is evident that water payments could be a hindering factor to get access to water. Those households only able to pay the water tariff will have access to water supply. This leads to water scarcity in the house and potentially unsafe water handling practices.

## 4. Conclusion

The prevalence of unsafe water handling practices of the study area was still significant, indicating most of the communities are still now prone to contamination of household storage water. Higher proportion of *E. coli* bacteria (44% from the source and 91% from household storage) had been reported in the water sample, indicating the majority of the rural population is at high risk of waterborne diseases. Water source protection found to be a necessary condition, but never be sufficient for the provision of safe water supply and in reduction of diarrheal diseases. Lower level of education, low monthly income, larger family size, frequency of water collection, presence of payments for water service, and prior knowledge of water treatment were the contributing factors to unsafe water handling practice.

### 4.1. Recommendations

Concerned stakeholders that work in water supply, hygiene, and sanitation intervention should promote safe water handling practices, and household water treatment methods to make water safer would be a worthy intervention to improve drinking water quality. Available water sources should be adequately protected and maintained to minimize the risk exposure from external contamination. Regular water quality testing and quality control mechanism for rural water supply system need to be in place to ensure the safety of drinking water supply. Provision of capacity building on education, income-generating activities, family planning, and creating awareness water quality to the community water caretakers and water committee is mandatory. Future research should be focused on assessing seasonal change on the quality of water sources.

## Figures and Tables

**Table 1:** Sociodemographic characteristics of households, in Boloso Sore woreda, Wolaita zone, Ethiopia, January 2019 (*n* = 545).

Variables	Response category	Frequency	Percentage
Education	No formal education	345	63.3
	Grades 1–4	72	13.2
	Grades 5–8	83	15.2
	High school and above	45	8.3
Monthly income (ETB)	≤500 Birr	469	86.1
	>500 Birr	76	13.9
Occupation	Farmer	42	7.7
	Merchant	42	7.7
	Housewife	461	84.6

**Table 2:** Water sources and handling practice in Boloso Sore woreda, Wolaita zone, Ethiopia, January 2019 (*n* = 545).

Variables with response categories		Frequency	Percentage
Water source	Shallow well	163	29.9
	Protected hand-dug well	123	22.6
	Springs	259	47.5
Distance of water source	<30 min	447	82
	31–60 min	98	18
Water collection container	Clay pot	5	0.9
	Jerry can	540	99.1
Duration of washing the container	Daily	532	97.6
	Every other day	10	1.8
	Once a week	3	0.6
Cover of collection container	Yes	462	84.8
	No	83	15.2
Collection per day	Once	124	22.8
	Twice	350	64.2
	Three times	71	13

**Table 3:** Water handling related to storage and usage by households in Boloso Sore woreda, Wolaita zone, Ethiopia, January 2019.

Variables	Response category	Frequency	Percentage
Type of water storage	Pot	69	12.7
	Barrel	5	0.9
	Jerry can	471	86.4
Cleaning of storage	Every time before collection	498	91.4
	Sometimes	47	8.6
Type of cleaning materials	Water only	139	25.5
	Soap	119	21.8
	Ash	62	11.4
	Plant leaves	194	35.6
	Others	31	5.7
Storage cover	Yes	413	75.8
	No	132	24.2
Water drawing	Pouring	493	90.5
	Dipping	52	9.5
Hand washing before drawing	Yes	368	67.5
	No	177	32.5
Placement of drinking cup	On the table	289	53
	On the floor	142	26.1
	Hung on the wall	114	20.9
Separate cup for drinking	Yes	373	68.4
	No	172	31.6
Cleanness of storage	Clean	340	62.4
	Not	205	37.6
Household water handling practice	Poor/unsafe	157	28.8
	Good/safe	388	71.2

**Table 4:** Water quality analysis results per water scheme and households (100 ml sample).

SN	Water source	CFU/100 ml of water					
		At the source	At HH1	At HH2	At HH3	At HH4	At HH5
1	HDW1 (Achurachigntabia)	4	50	11	6	14	10
2	HDW2 (Bassa Toga)	2	4	8	3	6	NA
3	HDW3 (Tokisakereshe)	0	0	12	6	0	NA
4	HDW4 (Chama Bassa)	11	15	17	5	50	NA
5	HDW5 (Chama Gataro)	0	6	12	4	8	NA
6	SW1 (Sore homba mamed)	0	0	3	50	21	6
7	SW2 (Sore homba sodanco)	0	5	4	12		NA
8	SW3 (Achura Alemu)	100	2	100	100	100	NA
9	SW4 (Chama Arado)	2	100	26	14	2	NA
10	SW5 (Bassa Unchamo)	2	0	5	6	12	8
11	SP1 (Sore homba bridge spring)	0	5	0	11	4	0
12	SP2 (Tokissa Wadu)	0	1	100	6	9	NA
13	SP3 (Afamabancha Mache)	16	23	24	46		NA
14	SP4 (Bassasumamo)	0	4	15	21	2	NA
15	SP5 (Chama bale)	0	4	100	8	12	NA
16	SP6 (Achurasosuwa)	0	4	1	8	6	2
17	SP7 (Dolla Ballale)	0	5	9	6	2	NA
18	SP8 (Dollakulle)	6	12	9	16	50	NA

**Table 5:** Water quality level for the sources and household storage.

Type of water source	*E. coli* level per 100 ml water sample					
	Excellent (A) (<1)	Acceptable (B) (1–10)	Unacceptable (C) (11–50)	Grossly contaminated (D) (51–100)	>100	Total sample
Protected hand-dug well	2 (40%)	2 (40%)	1 (20%)	0	0	5 (27.78%)
Shallow well	2 (40%)	2 (40%)	0	0	1 (20%)	5 (27.78%)
Protected spring	6 (75%)	1 (12.5%)	1 (12.5%)	0	0	8 (44.44%)
Total	10 (56%)	5 (28%)	2 (11%)		1 (5%)	18 (100%)
Household water storage quality level (*n* = 75)	7 (9%)	38 (51%)	21 (28%)	3 (4%)	6 (8%)	75 (100%)

Lloyd and Helmer (1991)—water quality risk category.

**Table 6:** Factors associated with water handling practice in Boloso Sore woreda, Wolaita zone, SNNPR, Ethiopia, February 2019.

Variables		Water handling		95% CI	
		Good	Poor	COR	AOR
Respondent	Father	18	12	1.00	1.00
	Mother	323	138	1.56 (0.73, 3.32)	3.27 (0.69, 15.58)
	Son	47	7	4.48 (1.52, 13.17)	1.30 (0.12, 14.31)
Age (years)	15–30	185	62	1.63 (0.86, 3.09)	1.09 (0.51, 2.34)
	31–45	170	77	1.20 (0.64, 2.27)	0.93 (0.45, 1.89)
	46–60	33	18	1.00	1.00
Marital status	Married	345	152	1.00	1.00
	Single	43	5	3.79 (1.47, 9.75)	4.49 (0.55, 36.40)
Education level	Illiterate	229	116	1.00	1.00
	Elementary	117	37	1.60 (1.04, 2.47)	1.36 (0.79, 2.35)
	≥High school	42	4	5.32 (1.86, 15.19)	**3.37 (1.03, 11.57)** ^*∗*^
Monthly income (ETB)	≤500	345	150	1.00	
	501–100	43	7	2.67 (1.17, 6.07)	**2.37 (1.96, 5.85)** ^*∗*^
Occupation	Farmer	27	15	0.77 (0.40, 1.50)	1.45 (0.37, 5.56)
	Merchant	38	3	5.45 (1.65, 17.96)	3.38 (0.89, 12.71)
	Housewife	323	139	1.00	1.00
Family number	≤5	181	49	1.93 (1.30, 2.85)	**1.81 (1.15, 2.83)** ^*∗*^
	>5	207	108	1.00	1.00
Water source	Shallow well	114	49	1.04 (0.68, 1.59)	1.01 (0.60, 1.68)
	Hand-dug well	95	28	1.52 (0.92, 2.49)	1.87 (0.99, 3.53)
	Spring	179	80	1.00	1.00
Amount of water	5–20 liters	230	82	1.33 (0.921.93)	0.93 (0.57, 1.51)
	21–50 liters	158	75	1.00	1.00
Water collection per day	Once	87	37	2.16 (1.18, 3.95)	1.43 (0.64, 3.21)
	Twice	264	86	2.82 (1.67, 4.77)	**2.88 (1.56, 5.33)** ^*∗*^
	Three times	37	34	1.00	1.00
Payment for water	Yes	300	131	0.68 (0.42, 1.10)	**0.44 (0.24, 0.84)** ^*∗*^
	No	88	26	1.00	1.00
Distance of water source	<30 min	308	139	0.50 (0.29, 0.86)	0.55 (0.30, 1.03)
	31–60 min	80	18	1.00	1.00
Type of cleaning materials	Water only	88	51	0.70 (0.44, 1.11)	**0.42 (0.24, 0.72)** ^*∗*^
	Soap	95	24	1.61 (0.93, 2.77)	1.51 (0.82, 2.79)
	Ash or others	67	26	1.05 (0.60, 1.81)	0.79 (0.43, 1.49)
	Plant leaves	138	56	1.00	1.00
Knowledge of water treatment	Yes	215	53	2.44 (1.66, 3.59)	**2.40 (1.52, 3.79)** ^*∗*^
	No	173	104	1.00	1.00

^*∗*^Significant at *P* < 0.05.

## Data Availability

The data used to support the findings of this study are available from the corresponding author upon request.
